# Quantitative Assessment of Liver Impairment in Chronic Viral Hepatitis with [^99m^Tc]Tc-Mebrofenin: A Noninvasive Attempt to Stage Viral Hepatitis-Associated Liver Fibrosis

**DOI:** 10.3390/medicina58101333

**Published:** 2022-09-23

**Authors:** Donatas Jocius, Donatas Vajauskas, Kipras Mikelis, Skirmante Jokubauskiene, Jolita Jakutiene, Kestutis Strupas, Algirdas E. Tamosiunas

**Affiliations:** 1 Faculty of Medicine, Vilnius University, LT-03101 Vilnius, Lithuania; 2 Department of Radiology, Medical Academy, Lithuanian University of Health Science Kauno Klinikos, LT-50161 Kaunas, Lithuania; 3 National Center of Pathology, LT-03101 Vilnius, Lithuania; 4 Centre of Hepatology, Gastroenterology and Dietology, Vilnius University Hospital Santaros Klinikos, LT-08661 Vilnius, Lithuania

**Keywords:** liver fibrosis, chronic liver disease, hepatobiliary scintigraphy, 99mTc-mebrofenin

## Abstract

*Background and objectives*—Chronic viral hepatitis B and C infections are one of the leading causes of chronic liver impairment, resulting in liver fibrosis and liver cirrhosis. An early diagnosis with accurate liver fibrosis staging leads to a proper diagnosis, thus tailoring correct treatment. Both invasive and noninvasive techniques are used in the diagnosis and staging of chronic liver impairment. Those techniques include liver biopsy, multiple serological markers (as either single tests or combined panels), and imaging examinations, such as ultrasound or magnetic resonance elastography. Nuclear medicine probes may also be employed in staging liver fibrosis, although the literature scarcely reports this. The purpose of our study was to investigate whether a dynamic liver scintigraphy with [^99m^Tc]Tc-mebrofenin has any value in staging or grading chronic liver damage. *Materials and Methods—We prospectively enrolled patients with chronic viral hepatitis B and C infection referred for liver biopsy. All patient underwent dynamic liver scintigraphy with 99mTc-mebrofenin prior to liver biopsy. Dynamic liver scintigraphy was performed immediately after intravenous tracer injection for 30 min scanning time. Multiple scintigraphy parameters were calculated (whole liver lobe and focal area time to peak (TTP), 30 min to peak ratio (30/peak), whole lobe and focal area slope index in 350 s (slope_350). Liver biopsy took place shortly after imaging. Results*—We found that many dynamic scintigraphic parameters are positively or negatively associated with different stages of liver fibrosis. The main parameters that showed most value are the ratio between 30 min and the peak of the dynamic curve (30/peak_dex (ratio)), and liver clearance corrected for body surface area and liver area (LCL_m^2^_dm^2^ (%/min/m^2^/dm^2^)). *Conclusions*—Our present study proves that conducting dynamic liver scintigraphies with [^99m^Tc]Tc-mebrofenin has potential value in staging liver fibrosis. The benefits of this method, including whole liver imaging and direct imaging of the liver function, provide an advantage over presently used quantitative imaging modalities.

## 1. Introduction

Chronic viral hepatitis B and C infections are one of the leading causes of chronic liver impairment. They result in liver fibrosis and liver cirrhosis, with an estimated burden of hepatitis B and hepatitis C infections affecting 15 million and 13 million people in the European Union, respectively [1]. Despite a wide variety of etiological factors, generally, liver fibrosis has a common pathophysiological pathway, similar to that of other organ systems [2]. Acute and chronic inflammation present in the tissue results in cell damage and the release of inflammatory mediators (integrins, vasoactive peptides, cytokines, growth factors, etc.), which, in turn, activate inflammatory cells such as lymphocytes, polymorphonuclear leukocytes, macrophages, and others. Activated inflammatory cells induce fibrogenic effector cells, including fibroblasts and myofibroblasts, that are responsible for the development of the fibrotic process, producing extracellular matrix components [2]. Stellate cells are the main effectors in hepatic fibrosis, transforming into myofibroblasts when a chronic liver injury is present and acting as a contractile element in the fibrotic scar [3].

Cellular and immunologic alterations work together to promote progressive extracellular matrix formation and downregulate its degradation. This abnormal tissue “healing” process begins within the portal tracts and graduates toward adjacent portal tracts as the tissue injury continues trapping liver cells within it, finally forming regenerative nodules–a representative feature of liver cirrhosis [4].

Hepatocytes also undergo cellular changes together with structural tissue alterations when long-lasting inflammation is present. 

There are many uptake transporters, such as organic cation transporters (OCT), organic anion transporters (OAT) and organic anion transporter polypeptides (OATP), and efflux transporters, such as P-glycoprotein, multidrug resistance-associated proteins (MRP), breast cancer-resistance proteins (BCRP), and others; uptake transporters are situated on the healthy hepatocyte basolateral membrane, and efflux transporters on the apical membrane. In the event of liver disease, the many alterations in gene expression and in receptor expression of basolateral and apical membranes result in the altered transport of drugs and metabolic substrates [5]. 

Altogether, these structural and molecular changes lead to a deterioration of liver functions and are associated with multiple liver fibrosis–or cirrhosis-related complications as the disease progresses.

In chronic viral hepatitis, the progression of liver fibrosis may take many years until clinical symptoms occur, also depending on virus type and subtype, time of infection onset, coinfections, and other factors [6,7,8].

Once the disease is present, a timely diagnosis and prompt management can prevent its further progression and complications, precluding unnecessary treatment in the low stages of the disease, especially when interferon-based therapy is used [8]. Moreover, in the era of novel, direct-acting antiviral therapy that is able to define fibrosis at different stages, there are diagnostic methods that can lead us to precise decisions regarding treatment [8]. 

Historically, the staging of liver fibrosis was performed by liver biopsy, which, despite being the only method directly evaluating liver tissue, has several well-known drawbacks, including its small sampling size (representing only 1/50,000 of total liver tissue, thus excluding the possibility to depict fibrosis heterogeneity in different liver areas), high interobserver and intraobserver variability in evaluating biopsy specimens, no possibility of sub-staging liver fibrosis, the invasiveness of the procedure, and the relatively high price of the procedure [9,10,11]. 

Many direct and non-direct laboratory tests are available both alone and as part of a variety of panels, from the simple AST/ALT ratios to more complex tests, such as Fibrotest^®^, Hepascore^®^, FIB4, and others. Although simple and non-invasive, these tests still lack precision and accuracy [12].

Quantitative imaging techniques, which are implemented into clinical practice for noninvasive staging of liver fibrosis, have several benefits possibly overcoming the limitations of liver biopsy. Ultrasound elastography (USE), including transient (TE), point shear wave (pSWE), and multidimensional shear wave elastography (2D-SWE) provide noninvasive imaging without ionizing radiation, have a relatively low cost, and are widely available [13,14]. Several limitations of ultrasound elastography techniques are operator dependence, difficulty comparing results between different vendors, a relatively small sample size, and patient-related technical issues (obesity, ascites, breath hold problems, and uncooperative patients) [11].

Magnetic resonance (MR) imaging with MR elastography, DWI, T1 relaxometry, and dynamic contrast-enhanced imaging are also able to quantitatively evaluate diffuse liver changes, sample a bigger region of interest (ROI) as compared to USE, and are reproducible between different vendors [11,12,15,16]. The drawbacks of MR are its relatively low availability, high price, the need of specific equipment, a technically challenging procedure, and patient-related failures, including general contraindication for MR (metal prosthesis, cardiac devices, claustrophobia) [11]. Moreover, USE and MRE represent non-direct changes in liver damage that evaluate biomechanical alterations, including liver elasticity and viscosity, rather than liver function [11,16,17].

The liver surface nodularity and liver segmental volume ratio measured on routine computed tomography (CT) are also promising techniques for quantitatively evaluating diffuse liver changes, even on previously performed scans, although the method’s validation is rather limited [11,18,19,20].

Functional imaging, employing a wide spectrum of different tracers, can also be used to quantify diffuse liver changes. Conventional nuclear medicine tracers such as [^99m^Tc]TcGSA (technecium-99m-glycosyl serum albumin), [^99m^Tc]Tc-mebrofenin (technecium-99m-2,2′-[[2-[(3-Bromo-2,4,6-trimethylphenyl)-amino]-2-oxoethyl]imino] bisacetic acid) showed potential value in the assessment of the functional change in liver parenchyma, which becomes altered in chronic viral hepatitis [21,22]. During the course of chronic liver disease, many alterations of cell membrane receptors, such as asialoglycoproteins and transporters (e.g., OATP receptors), are present; these features could theoretically be exploited through imaging [5].

Moreover, iminodiacetic acid derivates exploit the same transporters on the hepatocyte membrane as hepatospecific MR contrast agents, and both techniques show similar results in liver parenchymal function evaluation, although there are only scarce cases reported in the literature evaluating liver scintigraphy with [^99m^Tc]Tc-mebrofenin in CLD [15,21,23]. Altered glucose metabolism in different liver fibrosis stages can also be quantified using positron emission tomography (PET) with [^18^F]FFDG (fluorine-18–fluorodeoxyglucose). Different liver-to-background ratios were found to represent different stages of fibrosis, although these results need to be clarified in prospective studies [24]. 

Recently, another noninvasive approach using circulatory microRNA has drawn attention as a noninvasive marker of liver damage. Several studies invested various circulatory microRNA (miR-221, miR-542, miR-21, miR-122, and others) and found differences between healthy subjects, patients with chronic viral hepatitis C, and cases of hepatocellular carcinoma (HCC) [25,26]. Although these attempts show promise, prospective, multicenter trials are needed to further clarify the merits of this method and the means of implementing microRNA into routine practice.

## 2. Aim

The aim of this study is to test whether hepatobiliary scintigraphy with [^99m^Tc]Tc-mebrofenin has any value in evaluating the grade of liver fibrosis in patients with chronic viral hepatitis.

## 3. Materials and Methods

During the period from August 2018 to January 2020, we prospectively enrolled patients with chronic viral hepatitis B and C who were referred to our center for a liver biopsy as a staging procedure before the initiation of specific antiviral treatment, and agreed to participate in the clinical study by signing informed consent forms (all inclusion and exclusion criteria of the study are presented in Table 1). 

The study was approved by the Vilnius Regional Biomedical Research Ethics Committee (registered 13 December 2016, reg. no. 158200-16-877-386) and conducted according to the principles of the Helsinki Declaration. 

The study protocol was set as follows—a dynamic liver scintigraphy with [^99m^Tc]Tc-mebrofenin and a liver biopsy within 2 weeks after imaging. [^99m^Tc]Tc-mebrofenin (technecium-99m-2,2’-[[2-[(3-Bromo-2,4,6-trimethylphenyl)-amino]-2-oxoethyl]imino] bisacetic acid; Bridatec, GE Healthcare) was used for the dynamic liver scintigraphy imaging. A radiopharmaceutical was prepared under sterile conditions in a fume hood. The dynamic liver scintigraphy with [^99m^Tc]Tc-mebrofenin (hepatobiliary scintigraphy) examination was performed using a GE Infinia Hawkeye dual head SPECT/CT gamma camera (General Electric Healthcare, Milwaukee, WI, USA). The dynamic planar scintigraphy was performed in the supine position, including the heart and liver in the field of view, using a low-energy, high-resolution collimator (energy window 130–150 keV, matrix 64 × 64); acquisition began immediately after the intravenous injection of [^99m^Tc]Tc-mebrofenin (median activity 205.5 MBq (SD ± 14.15)) and continued for 30 min; the static planar scintigraphy was subsequently performed for 10 min of scanning time. 

Images were reconstructed using the GE Xeleris 2 workstation (General Electric Healthcare, Milwaukee, WI, USA). A geometric mean data set from anterior and posterior projections was used for further calculations.

Regions of interest (ROI) were drawn on both dynamic and planar images calculating dynamic and static tracer parameters. ROIs were drawn on the aorta at the level of the celiac trunk, representing input function, whole right and left liver lobes representing whole lobe tracer dynamics, and small focal ROIs on peripheral right and left liver lobes (representing small parenchyma areas). The same parenchymal ROIs were drawn on static images. 

**Figure 1:**
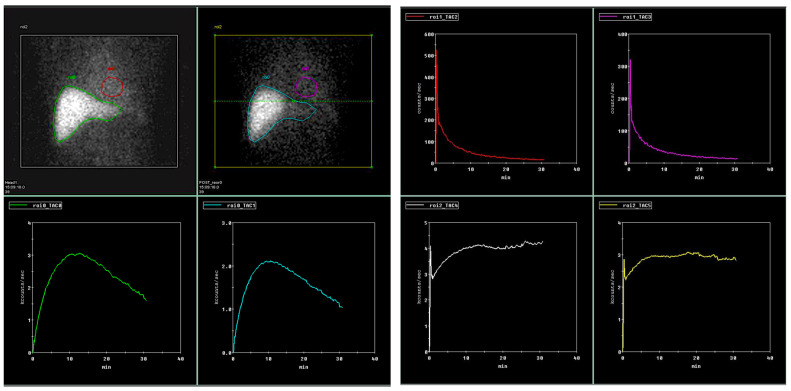
Liver clearance calculations from anterior and posterior dynamic liver scans. Green/electric blue curves representing liver area, red/pink curves representing heart area as an input function and white/yellow curves representing whole field of view. Calculations were made according to Ekman et al. [27].

When calculating liver clearance (LCL) (according to M. Ekman et al. [27]), the ROIs were drawn around the whole liver, the focal liver area positioned in the right liver lobe (representing biopsy area), the heart (representing input function), and whole field of view (representing total circulating tracer activity) (Figure 1).

In addition, parameters we corrected for body surface area (BSA), liver area (LA), and for acquiring independent results on both patient and liver areas.

Multiple dynamic scintigraphy parameters were obtained—time to peak (TTP; min.), time to half peak (halfTTP; min.) on both liver lobes and focal areas on the right and left liver lobes separately to represent tracer dynamics during the examination time of 30 min. Same ROIs were used for 30 min to peak ratio (30/peak; ratio) representing tracer retention inside the liver during excretion phase—the high ratio represents high tracer retention in the liver and low excretion.

The slope index (SLOPE; counts per second; c/s) was calculated during the uptake period from 150 to 350 s, representing tracer uptake by the hepatocytes during homogeneous tracer parenchymal distribution without any significant tracer excretion through the biliary tract. Since tracer uptake directly depends on the ROI’s size, we corrected the liver uptake rate for LA both on the whole liver and focal ROIs.

Liver clearance (LCL) was calculated according to Ekman et al. [27] and expressed in %/min representing [^99m^Tc]Tc-mebrofenin clearance from the blood by the hepatocytes. This measure was corrected for BSA (LCl_m^2^; %/min/m^2^) and for both BSA and LA (LCl_m^2^_dm^2^; %/min/m^2^/dm^2^). Liver clearance was also calculated in the focal area representing the biopsy sampling area (LCL_focal).

Several static parameters were also calculated—residual liver uptake measured at 30 min after tracer injection, drawing ROI on static geometric mean images of the whole liver and focal liver areas representing tracer retention in the liver after 30 min. 

A liver biopsy was performed within two weeks after the imaging took place. The liver biopsy procedure was performed on the right liver lobe by sampling 2–3 biopsy cores with a minimum of 3 cm length in total to acquire a representative sample with enough portal tracts needed for an accurate pathological examination. The METAVIR score evaluating liver fibrosis, the hepatitis activity index (HAI according to Ishak), and liver steatosis were evaluated during the pathological examinations by an experienced pathologist (S.J.) [28,29].

## 4. Statistical Analysis

The data were analyzed using Microsoft Excel (Microsoft Corporation, 2018) and IBM SPSS Statistics (Version 25.0. Armonk, NY, USA: IBM Corp.).

All variables were tested for normality using the Shapiro–Wilk test; statistical tests were selected accordingly and data were expressed in mean and standard error when normal data distribution was present, or in median and range if normal data distribution was not found. 

We looked for associations between the fibrosis score, hepatitis index, and steatosis grade from the core needle biopsy sample, with various scintigraphy parameters. The Student t test was used to look for differences between two groups of variables with normal distribution, and the Mann–Whitney U test for non-parametric continuous variables. For differences between three and more groups, a one-way analysis of variance (ANOVA) and the Kruskal–Wallis H test were used. Pearson’s correlation test and Spearman’s rank-order correlation coefficient were used to find correlations between continuous variables. A one-way analysis of covariance (ANCOVA) was used for differences between means, adjusted for the covariate. The significance level was set at 0.05. 

## 5. Results

### 5.1. Patient Characteristics

During the study period, 106 patients were invited to participate in a study; the study involved 72 patients who agreed to participate by signing an informed consent form and came for imaging studies. All 72 patients underwent scintigraphic liver imaging and only one did not come for the liver biopsy procedure due to non-related urgent comorbidity. 

The mean patient age was 45 years (range 18–80 years). 

Sixty-eight and four patients were infected with HCV and HBV infections, respectively. Nine patients were also coinfected with a HIV infection. The mean patient weight was 81.89 kg (range 48–130 kg), mean body mass index (BMI) was 26.83 kg/m^2^ (range 17.51–41.97), mean body surface area (BSA) was 1.98 m^2^ (range 1.45–2.52 m^2^). All patient data is presented in Table 2.

### 5.2. Histological Examination Results

Seventy-one patients underwent a liver biopsy procedure and were categorized into four grades according to METAVIR: fourteen patients—F1 (mild fibrosis), thirty-eight patients—F2 (significant fibrosis), twelve patients—F3 (advanced fibrosis), and seven patients—F4 (cirrhosis). Liver steatosis (mean 16.55%; range 0–75%) and hepatitis activity index (HAI) (median 5; range 3–9) were also measured.

### 5.3. Results of [^99m^Tc]Tc-Mebrofenin Scintigraphy

#### 5.3.1. Discriminating Mild Fibrosis (F1)

Several parameters were able to discriminate mild fibrosis from significant fibrosis and more including 30/peak_dex (0.62 vs. 0.73 (p 0.027)), 30/peak_dex_focal (0.59 vs. 0.7 (p 0.011)), 30/peak_sin_focal (0.58 vs. 0.7 (p 0.023)), and the area under the receiver operator characteristics (ROC) curve, which was 0.701, 0.718 and 0.709, respectively.

We also found a statistically significant difference in BSA and LA corrected liver clearance LCL_m^2^_dm^2^ (4.2%/min/m^2^/dm^2^ vs. 3.4%/min/m^2^/dm^2^; p 0.04), with AUROC 0.678.

Threshold values for the abovementioned parameters are presented in Table 3.

#### 5.3.2. Discriminating Mild–Significant Fibrosis (F1–F2)

Multiple measures significantly differentiated insignificant (F1–F2) versus significant fibrosis (F3–F4) (*p* value > 0.05): TTP_dex (12,11 min versus 15,14 min), 30/peak_dex (0.7 versus 0.85), 30/peak_sin (0.72 versus 0.86), TTP_dex_focal (12.54 min versus 14.61 min); TTP_sin_focal (12.54 min versus 16.88 min), 30/peak_dex_focal (0.66 versus 0.81), 30/peak_sin_focal (0.64 versus 0.82), SLOPE_dex (2.8 c/s versus 1.98 c/s), SLOPE_sin (1.82 c/s versus 1.22 c/s), SLOPE_dex_focal (3.99 c/s versus 2.72 c/s), SLOPE_sin_focal (2.91 c/s versus 2.08 c/s), LCL_m^2^ (8.7%/min/m^2^ versus 6.28%/min/m^2^), LCL_m^2^_dm^2^ (3.85%/min/m^2^/dm^2^ versus 2.82%/min/m^2^/dm^2^), LCL_focal_m^2^_dm^2^ (5.14%/min/m^2^/dm^2^ versus 3.73%/min./m^2^/dm^2^), and the area under receiver characteristics curve (AUROC) with a defined threshold. Sensitivities and specificities are presented in Table 4. 

#### 5.3.3. Discriminating Cirrhosis (F4)

Many scintigraphy parameters were also good discriminators between fibrosis and cirrhosis: TTP_dex (12.63 min versus 16.99 min), 30/peak_dex (0.73 versus 0.9), 30/peak_sin (0.73 versus 0.88), TTP_dex_focal (12.74 min versus 16.29 min); TTP_sin_focal (12.96 min versus 19.82 min), 30/peak_dex_focal (0.7 versus 0.87), 30/peak_sin_focal (0.68 versus 0.89), SLOPE_dex (2.71 c/s versus 1.39 c/s), SLOPE_sin (1.74 c/s versus 0.89 c/s), SLOPE_dex_focal (3.83 c/s versus 1.99 c/s), SLOPE_sin_focal (2.81 c/s versus 1.58 c/s), LCL_m^2^ (8.32%/min/m^2^ versus 5.35%/min/m^2^), LCL_m^2^_dm^2^ (3.76%/min/m^2^/dm^2^ versus 1.97%/min/m^2^/dm^2^), LCL_focal_m^2^_dm^2^ (4.99%/min/m^2^/dm^2^ versus 2.56%/min/m^2^/dm^2^), and the results of significant (*p* value > 0.05) parameters are presented in Table 5 with their representative threshold value, AUROC, sensitivity, and specificity.

Although at first glance tracer retention (residual uptake) was measured at 30 min after injection on static images and looked promising, at the end of the study we found no statistically significant difference in any of liver fibrosis grades.

### 5.4. Pathological Cofactors

Different liver steatosis levels and the hepatitis activity index (HAI) as a measure of inflammation activity may have had influence on different tracer kinetics, as they have an impact on liver stiffness measurements when using elastography techniques [30,31].

We performed a covariate analysis to check if liver steatosis and the HAI influence the results of dynamic liver scintigraphy with [^99m^Tc]Tc-mebrofenin at different liver fibrosis stages. There were no statistically significant differences regarding the HAI in any of the measured parameters. 

Liver steatosis, however, may have had an effect, since we found that differences in liver clearance (corrected both for liver area and body surface area) in mild–significant fibrosis groups (F1–2 vs. F3–4) and in the cirrhosis group (F1–3 vs. F4) were smaller when accounting for liver steatosis in the covariate analysis. This difference, although small, proved to be statistically significant (<0.05) (Table 6).

### 5.5. Liver Fibrosis Heterogeneity

The whole liver area and focal liver area clearance were calculated and also corrected for both BSA and LA, allowing a direct comparison between these two measurements; we found that there was a statistically significant difference–LCL_m^2^_dm^2^ 3.91%/min/m^2^/dm^2^ versus LCL_focal_m^2^_dm^2^ 4.84%/min/m^2^/dm^2^ (*p* value > 0.05). This result possibly represents liver fibrosis heterogeneity throughout the liver, since live fibrosis affects the liver irregularly. 

## 6. Discussion 

The stage of liver fibrosis is one of the cornerstone areas of interest before initiating the treatment of chronic viral hepatitis, as it bears information on the severity and prognostic information [32]. While liver biopsy remains the “gold standard” its well-known drawbacks stimulate scientists searching less invasive biomarkers.

Noninvasive serum biomarkers, both patented and freely available, are still lacking precision and are recommended to use only for patients at a high risk of advanced fibrosis, while the goal of the test itself is to rule out liver fibrosis rather than discover it [33,34]. A novel approach using plasma microRNA assays was proven to be capable of staging HBV- and HCV-associated liver fibrosis with high accuracy (AUROC over 80%) [35,36]. Combined microRNA panels were also used and proved as being able to separate significant fibrosis, though the study authors agreed that these findings should be further validated before implementing the method into clinical practice [36].

At present, quantitative imaging, such as USE and MRE, is used in a chronic liver disease setting to represent physical liver tissue changes during chronic liver disease, such as viral hepatitis, and these are the only imaging modalities selected by the guidelines [33]. The measurement of liver stiffness provides non-direct information regarding liver changes and liver function deterioration. In addition, several cofounding pathophysiological factors not related to CLD itself, such as inflammation, cholestasis, right heart failure, or hepatic venous congestion, together with different physiological patient states (fasting, breathing), may also alter liver stiffness irrespective of the liver disease stage [9,37].

CT imaging, which measured liver surface nodularity, blunt liver edge, parenchymal changes, and signs of portal hypertension, was also capable of separating cirrhotic liver tissue from healthy tissue, especially if combined with routine laboratory assays [18,20,38].

Nuclear medicine probes ([^99m^Tc]TcGSA, [^99m^Tc]Tc-mebrofenin) were previously used to assess liver functional changes preoperatively and prognosticate the future remnant liver tissue during major liver surgery [39,40,41,42]. One of the main benefits, also related to our study, is that nuclear medicine probes such as [^99m^Tc]Tc-mebrofenin measure liver function rather than its physical parameters, which also allows appreciating the quality of liver parenchyma, which is different in a healthy liver as opposed to cases of liver fibrosis [43]. These studies imply that probes such as these may also be used in CLD, and our present study results show that functional imaging with a liver-specific probe has value in quantitative liver evaluation, with several potential benefits regarding the direct testing of liver functional changes. In addition, these findings are supported by molecular studies showing that during the CLD course, the hepatocyte membrane structure undergoes changes, and many receptors and transporters are expressed differently. Moreover, the severity of these changes is also related to the severity of the disease [5,44]. 

To our knowledge, this is the first prospective, non-randomized study using a nuclear medicine probe in a CLD setting that evaluates different liver fibrosis stages.

We aimed at several goals regarding our study, and we sought to ascertain whether liver scintigraphy with [^99m^Tc]Tc-mebrofenin is related to liver damage in chronic viral hepatitis-associated liver fibrosis, and can separate different liver fibrosis stages, what are the parameters capable of doing so, and are there any new features that propose advantages over the already established limitations of imaging methods.

**Figure 2:**
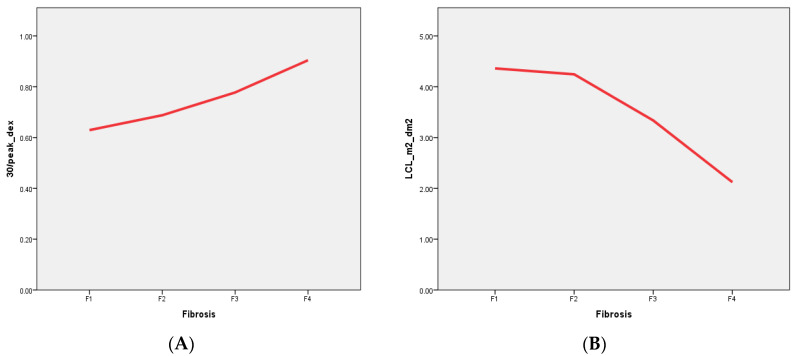
Correlation between the most significant scintigraphic parameters and liver fibrosis stages. (**A**) Direct positive correlation between liver excretion rate (30/peak) and liver fibrosis–tracer retention increases with increasing liver fibrosis. (**B**) Direct negative correlation between liver clearance (LCL) and liver fibrosis–tracer clearance decreases as liver fibrosis progress.

We found that dynamic hepatobiliary scintigraphy with [^99m^Tc]Tc-mebrofenin imaging-derived parameters have both direct positive (TTP, 30/peak) and negative (LCL) correlations with liver fibrosis stages in patients with liver fibrosis associated with chronic viral hepatitis (Figure 2). Moreover, by using liver scintigraphy with 99mTc–mebrofenin-driven parameters, we were able to stratify patients into four different liver fibrosis stages with high sensitivity and specificity (Table 7). 

**Figure 3:**
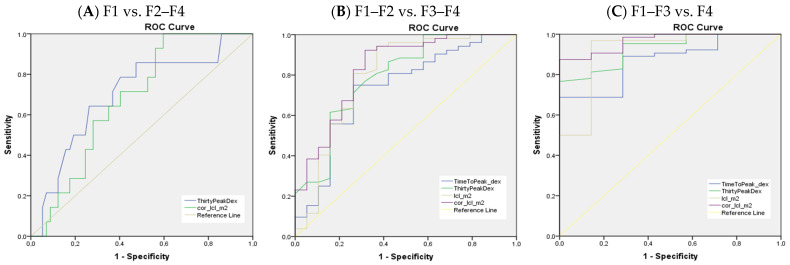
AUROC for most significant parameters discriminating liver fibrosis stages. ThirtyPeakDex–30/peak ratio in the right lobe. cor_lcl_m2–LCL_m^2^_dm^2^ (liver clearance corrected for BSA and LA). TimeToPeak_dex–TTP_dex.

The supporting data of these findings are based on the different numbers of functioning hepatocytes and their impaired function, with the altered hepatocyte membrane composition corresponding to the different stages of the disease [22,44]. A decreased number of functioning hepatocytes together with their altered membrane structure leads to prolonged tracer accumulation time in the liver (represented by TTP) and a prolonged tracer excretion from the liver parenchyma to the biliary system (represented by 30/peak, Figure 3A) [45]. Statistically significant differences were seen between different liver fibrosis categories (Table 3, Table 4 and Table 5).

[^99m^Tc]Tc-mebrofenin clearance (LCL), representing tracer clearance from the blood by the hepatocytes, is another measure that showed a direct inversed relation to liver disease severity—liver clearance decreased with increasing liver fibrosis stage (Figure 3B). This measurement, previously used by M. Ekman et al. [27] and later by R. J. Benink [40] and W. de Graaf [41,42], represents the total liver function, which clearly decreases with each higher stage of liver fibrosis.

The primary scope of this study posed the following question: is liver scintigraphy with [^99m^Tc]Tc-mebrofenin capable of staging patients with liver fibrosis?

Multiple parameters were good discriminators between the fibrosis stages, and while a few of them had statistical significance (excretion parameters namely 30/peak and LCL; Table 3) in discriminating mild liver fibrosis with increasing stages of fibrosis, many more of the scintigraphy parameters presented in Table 4 and Table 5 were good discriminators between significant and advanced fibrosis. 

**Figure 4:**
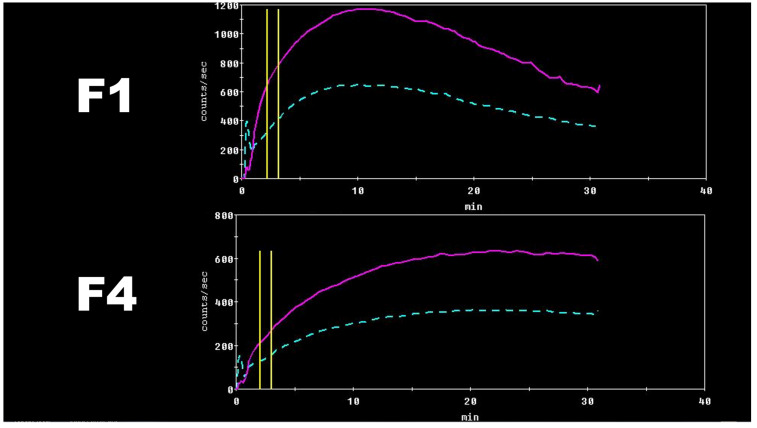
Two HCV patients, the first with F1 and the second with F4 fibrosis. There are clear differences between the tracer dynamics, including tracer acceleration (SLOPE, TTP) and excretion (30/peak). Calculated liver clearance was also different–F1 LCL_m^2^ 10.51%/min/m^2^, LCL_m^2^_dm^2^–5.41%/min/m^2^/dm^2^ and F4 LCL_m^2^ 4.64%/min/m^2^, LCL_m^2^_dm^2^–1.78%/min/m^2^/dm^2^.

These results can be theoretically explained by altered hepatocyte membrane composition during the CLD course and a decreased number of organic anions transporting polypeptides responsible for [^99m^Tc]Tc-mebrofenin internalization, all together with a decreased total amount of functioning hepatocytes [5,22,44]. Clear [^99m^Tc]Tc-mebrofenin dynamic differences can be seen in Figure 4.

Despite the multiple parameters calculated from [^99m^Tc]Tc-mebrofenin dynamic scintigraphy data, only a few were found as being statistically significant in all liver fibrosis categories—tracer excretion (30/peak_dex) and liver clearance (LCL_m^2^_dm^2^)—their data are presented in Figure 3 and Table 7. The sensitivity and specificity of these parameters increase with each increasing liver fibrosis stage (see Figure 2), similarly as with USE and MRE [46,47,48]. On the other hand, some studies of microRNA assays did show similar results in any fibrosis stage [35].

We calculated the threshold values for each parameter from the ROC curves; these results are presented in Table 3, Table 4 and Table 5. Conflicting results can be seen, since there are some notable differences between the same parameters in the same liver fibrosis category between the left and right liver lobes. A possible explanation could be the overlapping heterogeneity of liver fibrosis on planar images, and the overlapping of non-parenchymal structures–vessels, and gall bladder. Liver biopsies were performed by sampling only the right liver lobe and lateral subsegments (mainly S6 and S7); thus, the most accurate information is gathered from the right liver lobe only. Left liver lobe parameters should be interpreted cautiously, although the differences seen between the parameters of the right and left lobes may be related to different liver fibrosis stages, representing the heterogeneity of fibrosis.

## 7. Additional Findings

As reliable results were gathered from the right liver lobe, specifically from the focal area where liver biopsies were performed, we assumed that differences in other areas may represent different liver fibrosis stages, since liver fibrosis does not affect the liver homogeneously [49]. We tested this assumption by comparing focal liver clearance with whole liver clearance corrected for both BSA and LA and found statistically significant results (LCL_m^2^_dm^2^ 3.91%/min/m^2^/dm^2^ versus LCL_focal_m^2^_dm^2^ 4.84%/min/m^2^/dm^2^ (*p* value > 0.05)). As this was not the primary focus of the study, further studies on imaging liver heterogeneity with [^99m^Tc]Tc-mebrofenin are needed; however, the present results clearly indicate that this imaging method is capable of quantifying the whole liver and separate liver areas in one session.

Moreover, 99mTc–mebrofenin scintigraphy parameters such as the LCL represent the total functional capacity of the liver, allowing us to evaluate the total liver function—similar results were found by R. J. Benink [40] and W. de Graaf [41,42], which allowed separating good candidates for hepatectomy. 

Measuring total liver functional capacity in the CLD setting may overcome the limitations of the usually used non-direct quantification tools (USE and MRE), giving a holistic view of all liver tissue [47,48].

Special emphasis should be drawn to liver steatosis, since it is the only cofactor found in our study which may have an influence on calculating liver clearance (see Table 6).

Several technical strengths are clear in our present study. First, no technical failure was seen despite different patient ages, body mass, or other factors, which may at some point influence liver stiffness measurements performed using USE or MRE [47,48]. In addition, [^99m^Tc]Tc-mebrofenin scintigraphy is not related to some internal cofactors, such as elevated intraabdominal pressure or different vascular status (different blood flow), since it represents receptor-mediated tracer kinetics through the liver, making this technique more robust and not related to the varying status of the patient.

A handful of clinical benefits are also clear. Using [^99m^Tc]Tc-mebrofenin liver scintigraphy allows imaging of the whole liver, and from a clinical perspective, this gives a holistic overview regarding liver damage in contrary to liver biopsy, USE, and even MRE, where only limited liver areas of high confidence are used for measurement [11]. In addition, whole liver imaging allows the investigator to choose different and multiple areas to investigate, placing ROIs in addition to the whole liver ROI, and obtaining a profound understanding of total liver alterations during the disease course.

Although not tested by our study, [^99m^Tc]Tc-mebrofenin scintigraphy can be a valuable tool in longitudinal patient evaluation, because it represents liver function rather than liver stiffness as an indirect sign of liver function deterioration. Moreover, this imaging technique may be less related to internal pathological and physiological cofactors, but these theoretic assumptions should be evaluated in a prospective manner.

The price of [^99m^Tc]Tc-mebrofenin scintigraphy is rather small if compared to MRE. In our center single vial of pharmaceutical costs around EUR 28 and total cost of whole imaging including radiopharmaceutical preparation, imaging, and personal resources costs around EUR 100.

The relatively small sample size of our study should be viewed as a limitation, since larger studies may reveal more robust results on the acquired measurements. In addition, small separate liver fibrosis groups (for e.g., F4) should be viewed with caution and further addressed in another study with a higher cohort size.

Further studies are warranted, since many questions are still present—is this imaging method advantageous regarding treatment evaluation? Does it help make more informed treatment decisions and predict adverse events, such as CLD-related complications and liver cancer? In addition, a direct head-to-head comparison with the already implemented non-direct quantitative tools is needed to see if there are any benefits to this nuclear medicine imaging method.

Our study was conducted using planar imaging, and the measurements were made on a geometric mean data set, which may be less accurate than dynamic SPECT (volumetric) data; this should be confirmed by a prospective study employing the same tracer on dynamic SPECT.

## 8. Conclusions

Based on the results of our study, we can state that dynamic liver scintigraphy with [^99m^Tc]Tc-mebrofenin showed no failure and is an imaging modality able to stratify patients suffering from chronic viral hepatitis-related liver fibrosis. This imaging method provides several potential benefits over the presently used quantitative imaging modalities that measure direct liver function and produce images of the whole liver.

## Figures and Tables

**Table 1:** Inclusion and exclusion criteria of the study.

Inclusion Criteria	Exclusion Criteria
Adult age (18 or more)	Children
Treatment naïve chronic viral (HBV and HCV) hepatitis	Acute viral hepatitis
Compensated blood clotting parameters	Previously treated chronic viral hepatitis
Participation agreement	Impaired blood clotting parameter
	Disagreement to participate in the study

**Table 2:** Clinical characteristics of study population.

Variable	Value
Age (years)	45 years (18–80 years)
Weight (kg)	81.89 kg (48–130 kg)
BMI (kg/m^2^)	26.83 kg/m^2^ (17.51–41.97 kg/m^2^)
BSA (m^2^)	1.98 m^2^ (1.45–2.52 m^2^)
Virus type in patient population	
HBV	4 (5%)
HCV	68 (95%)
HIV coinfection	9 (12%)
Liver fibrosis stage (METAVIR)	
F1	14 (19%)
F2	38 (53%)
F3	12 (17%)
F4	7 (10%)
Liver steatosis	16.55% (0–75%)
HAI	5 (3–9)

**Table 3:** Dynamic scintigraphic quantitative parameters discriminating mild fibrosis (F1 vs. F2–F4).

Parameter	Threshold	AUROC	Sensitivity (%)	Specificity (%)	*p* Value
30/peak_dex	0.64	0.701	73	65	0.027
30/peak_dex_focal	0.65	0.718	70	79	0.011
30/peak_sin_focal	0.63	0.709	68	72	0.023
LCL_m^2^_dm^2^	3.76	0.678	71	60	0.04

**Table 4:** Dynamic scintigraphic quantitative parameters separating insignificant and significant liver fibrosis (F1–F2 vs. F3–F4).

Parameter	Threshold	AUROC	Sensitivity (%)	Specificity (%)	*p* Value
TTP_dex (min)	14.38	0.732	73	75	0.003
30/peak_dex	0.74	0.791	84	62	0.00001
30/peak_sin	0.79	0.798	63	83	0.00001
TTP_dex_focal (min)	12.04	0.685	84	50	0.015
TTP_sin_focal (min)	14.06	0.79	73	72	0.0009
30/peak_dex_focal	0.72	0.789	78	66	0.00001
30/peak_sin_focal	0.75	0.814	73	75	0.00002
SLOPE_dex (c/s)	2.32	0.807	68	87	0.00004
SLOPE_sin (c/s)	1.32	0.782	73	72	0.00001
SLOPE_dex_focal (c/s)	0.40	0.792	84	64	0.00001
SLOPE_sin_focal (c/s)	0.23	0.748	63	77	0.001
LCL_m^2^ (%/min/m^2^)	7.65	0.796	80	74	0.001
LCL_m^2^_dm^2^ (%/min/m^2^/dm^2^)	3.29	0.83	82	74	0.00003
LCL_focal_m^2^_dm^2^ (%/min/m^2^/dm^2^)	4.16	0.761	78	69	0.001

**Table 5:** Dynamic scintigraphic quantitative parameters discriminating cirrhosis (F1–F3 vs. F4).

Parameter	Threshold	AUROC	Sensitivity (%)	Specificity (%)	*p* Value
TTP_dex (min)	16.41	0.871	71	90	0.001
30/peak_dex	0.80	0.929	99	77	0.00001
30/peak_sin	0.85	0.884	71	91	0.001
TTP_dex_focal (min)	14.42	0.792	71	71	0.005
TTP_sin_focal (min)	15.42	0.922	99	79	0.00003
30/peak_dex_focal	0.78	0.887	85	77	0.001
30/peak_sin_focal	0.79	0.943	85	79	0.00002
SLOPE_dex (c/s)	2.03	0.977	99	94	0.00004
SLOPE_sin (c/s)	1.04	0.781	71	90	0.006
SLOPE_dex_focal (c/s)	0.32	0.914	85	85	0.0001
SLOPE_sin_focal (c/s)	0.20	0.794	71	83	0.01
LCL_m^2^ (%/min/m^2^)	5.57	0.915	96	86	0.0004
LCL_m^2^_dm^2^ (%/min/m^2^/dm^2^)	2.85	0.967	87	99	0.0005
LCL_focal_m^2^_dm^2^ (%/min/m^2^/dm^2^)	3.41	0.913	87	72	0.001

**Table 6:** Analysis of covariance for LCl_m2_dm2 by fibrosis with liver steatosis as the covariate.

Fibrosis	Mean (Unadjusted)	Std. Error	Mean (Adjusted for Steatosis)	Std. Error
F1–2	4.28	0.18	4.26	0.16
F3	3.34	0.24	3.33	0.33
F4	2.12	0.19	2.24	0.44

**Table 7:** Threshold of the most robust threshold values through liver stages.

	F1 vs. F2–F4	F1–F2 vs. F3–F4	F1–F3 vs. F4
**30/peak_dex (ratio)**	0.64	0.74	0.80
**LCL_m^2^_dm^2^ (%/min/m^2^/dm^2^)**	3.76	3.29	2.85

## Data Availability

The datasets used and/or analyzed during the current study are available from the corresponding author upon reasonable request.

## Abbreviations

2D-SWE	Multidimensional shear wave elastography
30/peak	30 min to peak ratio
ALT	Alanine amino transferase
AST	Aspartate amino transferase
AUROC	Area under receiver operator characteristics curve
BCRP	Breast cancer-resistance proteins
BMI	Body mass index
BSA	Body surface area
CLD	Chronic liver disease
CT	Computed tomography
FDG	Fluorodeoxyglucose
GSA	Glycosyl serum albumin
HAI	Hepatitis activity index
halfTTP	Time to half peak
LA	Liver area
LCL	Liver clearance
MR	Magnetic resonance
MRP	Multidrug resistance-associated proteins
OAT	Organic anion transporters
OATP	Organic anion transporter polypeptides
OCT	Organic cation transporters
PET	Positron emission tomography PET
pSWE	Point shear wave elastography
ROC	Receiver operator characteristics curve
ROI	Region of interest
SLOPE	Slope index
SPECT	Single photon emission computed tomography
TE	Transient elastography
TTP	Time to peak
USE	Ultrasound elastography
